# Supplementary material for: Prevalence of plantar ulcer and its risk factors in leprosy: a systematic review and meta-analysis
Source: J Foot Ankle Res. 2023 Nov 13;16:77. doi: 10.1186/s13047-023-00674-4 (PMC10641946; doi:10.1186/s13047-023-00674-4)
Supplement: Supplementary file 1 — Additional file 1. Protocol registration. [file 13047_2023_674_MOESM1_ESM.pdf]

Systematic review

A list of fields that can be edited in an update can be found here

1. * Review title.

Give the title of the review in English

Prevalence of neuropathic plantar ulcer and its risk factors in leprosy: a protocol for systematic review and meta-analysis

2. Original language title.

For reviews in languages other than English, give the title in the original language. This will be displayed with the English language title.

3. * Anticipated or actual start date.

Give the date the systematic review started or is expected to start.

01/02/2022

4. * Anticipated completion date.

Give the date by which the review is expected to be completed.

31/05/2022

5. * Stage of review at time of this submission.

This field uses answers to initial screening questions. It cannot be edited until after registration.

Tick the boxes to show which review tasks have been started and which have been completed.

Update this field each time any amendments are made to a published record.

The review has not yet started: No

Review stage	Started	Completed
Preliminary searches	Yes	No
Piloting of the study selection process	Yes	No
Formal screening of search results against eligibility criteria	Yes	No
Data extraction	No	No
Risk of bias (quality) assessment	No	No
Data analysis	No	No

Provide any other relevant information about the stage of the review here.

6. * Named contact.

The named contact is the guarantor for the accuracy of the information in the register record. This may be any member of the review team.

Karthikeyan Govindasamy

Email salutation (e.g. "Dr Smith" or "Joanne") for correspondence:

Mr Govindasamy

7. * Named contact email.

Give the electronic email address of the named contact.

karthik.govindasamy@warwick.ac.uk

8. Named contact address

Give the full institutional/organisational postal address for the named contact.

Warwick Medical School, Division of Health Sciences, University of Warwick, Coventry CV4 7AL, United Kingdom

9. Named contact phone number.

Give the telephone number for the named contact, including international dialling code.

+91 9935284315

10. * Organisational affiliation of the review.

Full title of the organisational affiliations for this review and website address if available. This field may be completed as 'None' if the review is not affiliated to any organisation.

University of Warwick

Organisation web address:

https://warwick.ac.uk/

11. * Review team members and their organisational affiliations.

Give the personal details and the organisational affiliations of each member of the review team. Affiliation refers to groups or organisations to which review team members belong. **NOTE: email and country now MUST be entered for each person, unless you are amending a published record.**

Mr Karthikeyan Govindasamy. University of Warwick
Dr Joydeepa Darlong. The Leprosy Mission Trust India
Dr Samuel Watson. University of Birmingham
Professor Paramjit Gill. University of Warwick

12. * Funding sources/sponsors.

Details of the individuals, organizations, groups, companies or other legal entities who have funded or sponsored the review.

This review is part of the PhD program under the project titled "Transforming the Treatment and Prevention of Leprosy and Buruli ulcers in Low and Middle-Income Countries (LMICs)" a multi-country project funded by the National Institute of Health Research, UK

Grant number(s)

State the funder, grant or award number and the date of award

NIHR200132

13. * Conflicts of interest.

List actual or perceived conflicts of interest (financial or academic).

None

14. Collaborators.

Give the name and affiliation of any individuals or organisations who are working on the review but who are not listed as review team members. **NOTE: email and country must be completed for each person, unless you are amending a published record.**

15. * Review question.

State the review question(s) clearly and precisely. It may be appropriate to break very broad questions down into a series of related more specific questions. Questions may be framed or refined using PI(E)COS or similar where relevant.

What is the prevalence of neuropathic plantar ulcer in the foot among people affected by leprosy? And what are the risk factors for development of neuropathic plantar ulcers among people affected by leprosy?

16. * Searches.

State the sources that will be searched (e.g. Medline). Give the search dates, and any restrictions (e.g. language or publication date). Do NOT enter the full search strategy (it may be provided as a link or attachment below.)

We will perform searches in the following databases MEDLINE (using Ovid platform), EMBASE (using Ovid platform), Web of Science, CINAHL, BVS (Biblioteca Virtual de Saude), and INFOLEP. In addition, we will look for unpublished resources like conference abstracts and conference proceedings which reported prevalence of ulcers. Additionally, we will screen reference lists of identified studies to be included in the review. We will use two keywords and its related terms ‘leprosy’ and ‘ulcers’ in the search strategies combining medical subject headings (MeSH) and keywords. We will include studies from January 1990 to July 2021. No restriction to language or scientific journals will be considered.

17. URL to search strategy.

Upload a file with your search strategy, or an example of a search strategy for a specific database, (including the keywords) in pdf or word format. In doing so you are consenting to the file being made publicly accessible. Or provide a URL or link to the strategy. Do NOT provide links to your search **results**.

https://www.crd.york.ac.uk/PROSPEROFILES/316726_STRATEGY_20220314.pdf

Alternatively, upload your search strategy to CRD in pdf format. Please note that by doing so you are consenting to the file being made publicly accessible.

Do not make this file publicly available until the review is complete

18. * Condition or domain being studied.

Give a short description of the disease, condition or healthcare domain being studied in your systematic review.

Leprosy or Hansen's disease. The plantar ulcers are common and serious complications in leprosy. Understanding the prevalence and risk factors for plantar ulcers will help in planning effective intervention of its management.

19. * Participants/population.

Specify the participants or populations being studied in the review. The preferred format includes details of both inclusion and exclusion criteria.

Review population consists of person affected by leprosy with loss of sensation in the foot with or without neuropathic ulcer will be included in the review.

20. * Intervention(s), exposure(s).

Give full and clear descriptions or definitions of the interventions or the exposures to be reviewed. The preferred format includes details of both inclusion and exclusion criteria.

Not specific – interventions related to prevention of neuropathic ulcers / secondary impairments in leprosy through self-care program or protective footwear.

21. * Comparator(s)/control.

Where relevant, give details of the alternatives against which the intervention/exposure will be compared (e.g. another intervention or a non-exposed control group). The preferred format includes details of both inclusion and exclusion criteria.

None.

22. * Types of study to be included.

Give details of the study designs (e.g. RCT) that are eligible for inclusion in the review. The preferred format includes both inclusion and exclusion criteria. If there are no restrictions on the types of study, this should be stated.

We will include studies of any design that report a point prevalence of neuropathic plantar ulcer and its risk factors.

23. Context.

Give summary details of the setting or other relevant characteristics, which help define the inclusion or exclusion criteria.

We will include studies that include prevalence of ulcers measured at the time of diagnosis, during the chemotherapy for leprosy and after completion of chemotherapy.

24. * Main outcome(s).

Give the pre-specified main (most important) outcomes of the review, including details of how the outcome is defined and measured and when these measurement are made, if these are part of the review inclusion criteria.

Prevalence: (a) Point prevalence of neuropathic ulcer among patients with loss of sensation in their feet due to leprosy (b) Point prevalence of neuropathic ulcer among people affected by leprosy (with or without neuropathy).Risk factors: Risk factors (causative and predictive) for development of neuropathic ulcer in foot with loss of sensation. The major factors included are loss of sensation in the sole of the foot with or without claw toes, previous ulcer, high plantar pressure, foot drop, gross damage to bones of the joint of the foot, higher vibration perception threshold, callus/cracks, non-adherence to wearing footwear, non-adherence to self-care, higher level of disability, age, gender and family support.

Measures of effect

Please specify the effect measure(s) for you main outcome(s) e.g. relative risks, odds ratios, risk difference, and/or 'number needed to treat.

Not applicable.

25. * Additional outcome(s).

List the pre-specified additional outcomes of the review, with a similar level of detail to that required for main outcomes. Where there are no additional outcomes please state 'None' or 'Not applicable' as appropriate to the review

None.

Measures of effect

Please specify the effect measure(s) for you additional outcome(s) e.g. relative risks, odds ratios, risk difference, and/or 'number needed to treat.

26. * Data extraction (selection and coding).

Describe how studies will be selected for inclusion. State what data will be extracted or obtained. State how this will be done and recorded.

Study selection and data extraction will be done by two authors (KG and JD) independently. The results of all searches will be saved using reference manager software (EndNote 20) to identify and remove duplicates. The final list of search results will be screened after reviewing the title first to be included for abstract review. All studies that are suitable at abstract review will be included for full text review. PG and SW will oversee the process of selection of studies, data extraction and analysis and any discrepancies in selection of studies and data extraction. Disagreement between two independent authors will be resolved by verification and consensus.

The extraction will be done on a standardized checklist in MS excel spreadsheet. For the first outcome, prevalence of neuropathic ulcer, the data extraction checklist will include author name, year of publication, region (the area where the study was conducted), study setting (hospital/community), rural area/urban area, study design, sample size, study population, number of patients with loss of sensation in the foot and number of participants with outcome (ulcer) For the second outcome, we will extract relevant data for narrative synthesis using quantitative data on risk factor for ulcer development which will be categorized as causative and predictive variables.

27. * Risk of bias (quality) assessment.

State which characteristics of the studies will be assessed and/or any formal risk of bias/quality assessment tools that will be used.

Quality of included studies will be assessed using the risk of bias tools for prevalence studies. To assess quality for studies of nonrandomized study designs such as before-after studies, cohort studies and case-control studies (only for risk factor part of the review), we will use Risk of Bias Assessment tool for Nonrandomized Studies (RoBANS). Only those studies with low risk of bias will be considered adequate quality to be included in the analysis. The risk of bias assessment will be done by KG and JD independently.

28. * Strategy for data synthesis.

Describe the methods you plan to use to synthesise data. This **must not be generic text** but should be **specific to your review** and describe how the proposed approach will be applied to your data. If meta-analysis is planned, describe the models to be used, methods to explore statistical heterogeneity, and software package to be used.

We will conduct a random-effects meta-analysis to estimate the pooled point prevalence of neuropathic ulcer in the two populations (in those with neuropathy and in all patients affected by leprosy). We will estimate the I^2 statistic to examine between-study heterogeneity. As the prevalence is a value between zero and one, a model assuming a normal distribution may be inappropriate, we will therefore use a Beta-Binomial meta-analysis model. We will conduct a narrative synthesis of quantitative data on risk factors for development of neuropathic plantar ulcer. We anticipate heterogeneity between studies in their methodological, statistical, and clinical approaches, hence the narrative synthesis approach. For example, the plantar pressure may be reported as continuous variable as peak plantar pressure or as binary/ordinal variables as low, normal and high as compared to normal foot or foot with loss of sensation but without ulcer. Similarly, the loss of sensation in the foot may be ascertained using monofilament or ball-point pen. These risk factors may be reported from unadjusted or adjusted models and from heterogeneous patient groups. We will stratify and combine the studies based on risk factors, population, and method of analysis. We will then broadly categorize the risk factors into two broad groups as predictive and causative factors and narrate the association between these factors with the outcome (ulcer) to synthesis evidence. We will also explore and identify mediator variables between risk factors and ulcer, for example the non-adherence to protective footwear which can be the intermediary variable between the loss of sensation in the sole and ulcer. Based on the narrative synthesis of risk factors we will make a causal diagram to visually articulate the causal pathways for development of neuropathic plantar ulcer in leprosy.

29. * Analysis of subgroups or subsets.

State any planned investigation of ‘subgroups’. Be clear and specific about which type of study or participant will be included in each group or covariate investigated. State the planned analytic approach.

For prevalence of plantar ulcers, where sufficient studies, i.e., at least 2 from different regions, are available we will report the prevalence according to region as recognized by World Health Organization as sub-group analysis.

30. * Type and method of review.

Select the type of review, review method and health area from the lists below.

Type of review

Cost effectiveness

No

Diagnostic

No

Epidemiologic

No

Individual patient data (IPD) meta-analysis

No

Intervention

No

Living systematic review

No

Meta-analysis

Yes

Methodology

No

Narrative synthesis

No

Network meta-analysis

No

Pre-clinical

No

Prevention

No

Prognostic

No

Prospective meta-analysis (PMA)

No

Review of reviews

No

Service delivery

No

Synthesis of qualitative studies

No

Systematic review

Yes

Other

No

Health area of the review

Alcohol/substance misuse/abuse

No

Blood and immune system

No

Cancer

No

Cardiovascular

No

Care of the elderly

No

Child health

No

Complementary therapies

No

COVID-19

No

Crime and justice

No

Dental

No

Digestive system

No

Ear, nose and throat

No

Education

No

Endocrine and metabolic disorders

No

Eye disorders

No

General interest

No

Genetics

No

Health inequalities/health equity

No

Infections and infestations

No

International development

No

Mental health and behavioural conditions

No

Musculoskeletal

No

Neurological

No

Nursing

No

Obstetrics and gynaecology

No

Oral health

No

Palliative care

No

Perioperative care

No

Physiotherapy

No

Pregnancy and childbirth

No

Public health (including social determinants of health)

No

Rehabilitation

No

Respiratory disorders

No

Service delivery

No

Skin disorders

No

Social care

No

Surgery

No

Tropical Medicine

No

Urological

No

Wounds, injuries and accidents

Yes

Violence and abuse

No

31. Language.

Select each language individually to add it to the list below, use the bin icon to remove any added in error.

English

There is an English language summary.

32. * Country.

Select the country in which the review is being carried out. For multi-national collaborations select all the countries involved.

India

33. Other registration details.

Name any other organisation where the systematic review title or protocol is registered (e.g. Campbell, or The Joanna Briggs Institute) together with any unique identification number assigned by them. If extracted data will be stored and made available through a repository such as the Systematic Review Data Repository (SRDR), details and a link should be included here. If none, leave blank.

34. Reference and/or URL for published protocol.

If the protocol for this review is published provide details (authors, title and journal details, preferably in Vancouver format)

Add web link to the published protocol.

Or, upload your published protocol here in pdf format. Note that the upload will be publicly accessible.

No I do not make this file publicly available until the review is complete

Please note that the information required in the PROSPERO registration form must be completed in full even if access to a protocol is given.

35. Dissemination plans.

Do you intend to publish the review on completion?

Yes

Give brief details of plans for communicating review findings.?

The review findings will be published in the open access peer reviewed journal.

36. Keywords.

Give words or phrases that best describe the review. Separate keywords with a semicolon or new line. Keywords help PROSPERO users find your review (keywords do not appear in the public record but are included in searches). Be as specific and precise as possible. Avoid acronyms and abbreviations unless these are in wide use.

Leprosy; plantar ulcers; loss of sensation in the foot

37. Details of any existing review of the same topic by the same authors.

If you are registering an update of an existing review give details of the earlier versions and include a full bibliographic reference, if available.

38. * Current review status.

Update review status when the review is completed and when it is published.New registrations must be ongoing so this field is not editable for initial submission.

Please provide anticipated publication date

Review_Ongoing

39. Any additional information.

Provide any other information relevant to the registration of this review.

40. Details of final report/publication(s) or preprints if available.

Leave empty until publication details are available OR you have a link to a preprint (NOTE: this field is not editable for initial submission). List authors, title and journal details preferably in Vancouver format.

Give the link to the published review or preprint.
