# Supplementary material for: Prevalence of plantar ulcer and its risk factors in leprosy: a systematic review and meta-analysis
Source: J Foot Ankle Res. 2023 Nov 13;16:77. doi: 10.1186/s13047-023-00674-4 (PMC10641946; doi:10.1186/s13047-023-00674-4)
Supplement: Supplementary file 3 — Additional file 3. Search strategy. [file 13047_2023_674_MOESM3_ESM.pdf]

Search Strategy

Medline (Ovid) search strategy

Ovid MEDLINE(R) ALL <1946 to June 17, 2021>

1 lepro*.mp. or (Leprosy/ or Leprosy, Borderline/ or Leprosy, Lepromatous/ or Leprosy, Multibacillary/ or Leprosy, Paucibacillary/ or Leprosy, Tuberculoid/) [mp=title, abstract, original title, name of substance word, subject heading word, floating sub-heading word, keyword heading word, organism supplementary concept word, protocol supplementary concept word, rare disease supplementary concept word, unique identifier, synonyms] 27194

2 lepra*.mp. 9764

3 hansen*.mp. 6438

4 1 or 2 or 3 34084

5 exp Skin Ulcer/ or exp Ulcer/ or ulcer*.mp. 274329

6 4 and 5 1140

7 exp Buruli Ulcer/ or buruli*.mp. 1128

8 6 not 7 1005

9 exp Foot Diseases/ or exp Foot/ or exp Foot Deformities/ or exp Foot Ulcer/ or exp Foot Deformities, Acquired/ 81923

10 (foot or feet or plantar).mp. [mp=title, abstract, original title, name of substance word, subject heading word, floating sub-heading word, keyword heading word, organism supplementary concept word, protocol supplementary concept word, rare disease supplementary concept word, unique identifier, synonyms] 160621

11 9 or 10 176541

12 8 and 11 414

13 limit 12 to yr="1990 -Current" 238

Embase (OVID) search strategy

Embase Classic+Embase <1947 to 2021 Week 23>

1 exp leprosy/ or lepro*.mp. or exp lepromatous leprosy/ 37498
2 exp leprosy/ or exp borderline leprosy/ or lepra*.mp. or exp lepromatous leprosy/ 37029
3 hansen*.mp. 9463
4 1 or 2 or 3 47018
5 exp skin ulcer/ or exp ulcer incidence/ or ulcer*.mp. or exp foot ulcer/ or exp ulcer/ or exp trophic ulcer/ or exp ulcer perforation/ or exp ulcer prevention device/ or exp plantar ulcer/ 476820
6 4 and 5 2657
7 Buruli ulcer/ or buruli.mp. 1468
8 6 not 7 2392
9 exp foot ulcer/ or foot.mp. or exp foot orthosis/ or exp foot/ or exp foot care/ or exp foot disease/ 254360
10 (foot or feet or plantar).mp. [mp=title, abstract, heading word, drug trade name, original title, device manufacturer, drug manufacturer, device trade name, keyword, floating subheading word, candidate term word] 242744
11 9 or 10 292209
12 8 and 11 729
13 limit 12 to yr="1990 -Current" 430

Web of science search strategy

#1 TS=(lepros*) OR TS=(hansen*) 23633
#2 TS=(ulcer*) OR TS=(skin ulcer) OR TS=(ulcer) OR TS=(plantar ulcer) OR TS=(foot ulcer) 184067
#3 #2 AND #1 623
#4 TS=(Buruli OR Buruli ulcer) 1121
#5 #3 NOT #4 491

CINAHL (EBSCO) search strategy

Saturday, June 19, 2021 10:14:13 AM

#	Query	Limiters/Expanders	Last Run Via	Results
S5	#S4 NOT #S3	Expanders - Apply equivalent subjects Search modes - Find all my search terms	Interface - EBSCOhost Research Databases Search Screen - Advanced Search Database - eBook Collection (EBSCOhost);CINAHL	160
S4	S1 AND S2	Expanders - Apply equivalent subjects Search modes - Find all my search terms	Interface - EBSCOhost Research Databases Search Screen - Advanced Search Database - eBook Collection (EBSCOhost);CINAHL	173
S3	TI buruli ulcer* OR AB buruli ulcer*	Limiters - Published Date: 19900101-20210631 Expanders - Apply equivalent subjects Search modes - Find all my search terms	Interface - EBSCOhost Research Databases Search Screen - Advanced Search Database - eBook Collection (EBSCOhost);CINAHL	156
S2	TI ulcer* OR AB ulcer* OR TI skin ulcer OR AB skin ulcer OR TI foot ulcer OR AB foot ulcer OR TI ulcer prevention* OR AB ulcer prevention* OR TI (foot or feet or lower limb or lower extremities) OR AB (foot or feet or lower limb or lower extremities) OR TI foot disease OR AB foot disease	Limiters - Published Date: 19900101-20210631 Expanders - Apply equivalent subjects Search modes - Find all my search terms	Interface - EBSCOhost Research Databases Search Screen - Advanced Search Database - eBook Collection (EBSCOhost);CINAHL	107,066
S1	TI (leprosy or hansen's disease) OR AB (leprosy or hansen's disease) OR TI hansen's disease OR AB hansen's disease OR TI hansen* OR AB hansen*	Limiters - Published Date: 19900101-20210631 Expanders - Apply equivalent subjects Search modes - Find all my search terms	Interface - EBSCOhost Research Databases Search Screen - Advanced Search Database - eBook Collection (EBSCOhost);CINAHL	2,130

BVS (Biblioteca Virtual de Saude) search strategy

(lepros*) OR (hansen*) AND (ulcer*) AND (foot diseases) AND NOT (buruli) AND (mj:("Leprosy" OR "Foot Diseases" OR "Skin Ulcer" OR "Foot Ulcer" OR "Ulcer" OR "Shoes" OR "Peripheral Nervous System Diseases" OR "Foot" OR "Leprosy, Lepromatous" OR "Foot Deformities, Acquired" OR "Leprosy, Borderline" OR "Leprosy, Tuberculoid" OR "Sensation" OR "Tibial Nerve" OR "Nervous System Diseases" OR "Podiatry" OR "Pressure" OR "Recurrence" OR "Sensation Disorders" OR "Leprosy, Multibacillary" OR "Neuritis" OR "Orthotic Devices" OR "Skin Diseases")) AND (year_cluster:[1990 TO 2021])
Total hits 59

Search strategy for INFOLEP database

Outcome	Search strategy
Prevalence of neuropathic ulcer	“Prevalence foot ulcer + filter on NTD: leprosy
Risk factors for neuropathic ulcer	“Risk factors plantar ulcer”<br>“Predictor plantar ulcer”<br>“Cause plantar ulcer”<br>“Risk factors plantar ulcer” + Filter on NTD: leprosy<br>“Predictor plantar ulcer” + Filter on NTD: leprosy
