# Supplementary material for: Prevalence of plantar ulcer and its risk factors in leprosy: a systematic review and meta-analysis
Source: J Foot Ankle Res. 2023 Nov 13;16:77. doi: 10.1186/s13047-023-00674-4 (PMC10641946; doi:10.1186/s13047-023-00674-4)
Supplement: Supplementary file 4 — Additional file 4. List of excluded studies. [file 13047_2023_674_MOESM4_ESM.pdf]

List of studies excluded after screening full text (n=47)

A. Full text report not available & other than English language (n=6)

1. Ramarorazana S, Rene JP, Schwartzl E, Randrianomenjanahary J, Razafindramboa H, Di Schino M. One-year follow-up of 466 nerve decompression procedures in 123 lepers during multidrug therapy in Madagascar. [French]. Med Trop (Mars). 1995;55(2):146-50.
2. Grauwin MY, Ndiaye A, Sylla PM, Gaye AB, Mane I, Cartel JL, et al. [Can plantar ulcers associated with leprosy be treated in the field. Results of experience in Senegal]. Sante. 1998;8(3):199-204.
3. Park SK, Lim JH. Clinical characteristics of Sorokdo's Hansen patients having neuropathic ulcer on the plantar region. Korean Leprosy Bulletin. 2011;44(1):27-34.
4. Brito KKG, Soares MJlGO, Costa MML, Oliveira SHS. PRÁTICAS E LIMITAÇÕES DE CLIENTES COM HANSENÍASE NO CUIDAR DAS LESÕES CUT¶NEAS. Journal of Nursing UFPE / Revista de Enfermagem UFPE. 2014;8(1):16-21.
5. BİÇEr GenÇBay M, KÜLtÜR A, Aydin ÖZÜÇEÜK Ş, ÖZÜÇElİK DN. Lepra Hastalarındaki El, Ayak ve Göz Bozuklukları. Turkiye Klinikleri Journal of Medical Sciences. 2019;39(1):83-8.
6. de Medeiros Nóbrega M, Gonçalves de Brito KK, Missias Villaverde Antas E, Soares Carvalho P, Malzac Freire de Santana E, Alves da Silva M, et al. SELF-CARE IN INDIVIDUALS WITH LEPROSY: EVALUATING PRACTICES IN THE SECONDARY HEALTH CARE NETWORK. Cogitare Enfermagem. 2020;25:194-204.

B. Other than English language (n=6)

7. Vulliet F, Tschibangu P. [Treatment and rehabilitation of disabilities caused by leprosy at a rural hospital (Kapolowe, Zaire)]. Acta Leprologica. 1990;7(3):225-8.
8. Hirzel C, Grauwin MY, Mane I, Cartel JL. Results obtained by a mobile disability prevention unit at the Institut de Leprologie (Institute of Leprosy Research), Dakar. [French]. Acta Leprologica. 1995;9(4):183-6.
9. Mane I, Grauwin MY, Cartel JL. Frequency of chronic plantar ulcers in leprosy patients according to treatment by dapsone monotherapy or multidrug therapy. [French]. Acta Leprologica. 1995;9(3):127-31.
10. Tiendrebeogo A, Djakeaux DS, Asse H, Eba ME, Sica A. A survey of leprosy disabilities among patients treated with MDT in Ivory Coast. [French]. Acta Leprologica. 1997;10(3):151-8.
11. Kim PJ, Kim SY. The possible predicting factor of the plantar ulcer in patients with leprosy. Korean Leprosy Bulletin. 2008;41(2):23-35.
12. Malzac Freire de Santana E, Gonçalves de Brito KK, Missias Villaverde Antas E, Sgren da Costa Andrade S, Vitorino Diniz I, de Macêdo Lima S, et al. Características sociodemográficas e clínicas da hanseníase: um estudo populacional. Enfermagem Brasil. 2018;17(3):227-35.

C. Conference abstract – incomplete data (n=6)

13. Patra P, Som AK, Manglani PR. Prevention of recurrence of ulcers in persons affected by leprosy. Indian Journal of Leprosy. 2012;84 (1):109.
14. Prakash D, Ebenezer M. "The importance of plantar soft tissue resilience and plantar padding in the genesis of plantar ulcers in leprosy". Indian Journal of Leprosy. 2012;84 (1):97-8.
15. Ranjan LN, Kanti DN, Nath CS, Chandra GR. Grade 2 deformities in leprosy: Evaluation of its clinico-demographic association. Indian Journal of Leprosy2012. p. 99.

16. Tiwari SN, Pandey SC, Chandra U. Disability control by self-care at home in Bihar. Indian Journal of Leprosy2012. p. 108-9.
17. Mishra SK, Singh RK, Singh A, Srivastava GC, Singh L, Kumar S. Integrated prevention of disability programme for disabled person due to leprosy and elephantiasis in Bihar, India. Indian Journal of Leprosy; October-December2017. p. 280-1.
18. Pai VV, Dhamale CK, Kshirsagar T, Kute A, Project BL, Rokade V. Assessment and impact of disability care services-observations from referral center. Indian Journal of Leprosy; October-December2017. p. 278.

D. Denominator not available (n=8)

19. Krishnamoorthy KV, Santaram V, Behera M, Pramanik JL. Incidence of plantar ulcers in Hansen's disease (HD) in R.L.T. and R.I., Aska. One year study. Star. 1991;51(2):8-9+16.
20. Li JL, Mu HJ, Ke W, Bao X, Wang Y, Shen LM, et al. Government health workers as implementers of prevention of disability measures: an assessment of a prevention of disability project in selected counties of Guizhou Province, Peoples' Republic of China. Leprosy Review. 2008;79(3):295-302.
21. Nair SP, Mathew R. Grade 2 disability in leprosy: Scenario in the post-elimination phase of leprosy from a tertiary care center. Indian Journal of Leprosy. 2017;89(3):127-37.
22. Uikey D, Joshi R, Shah B, Verma N. Leprosy scenario in Ahmedabad District (Gujarat). Indian J Dermatol. 2019;64(5):383-8.
23. Cakiner T, Yuksel A, Senal Egit A, Cagri G, Karacorlu M, Kultur A. The extent of leprosy-related disabilities in Istanbul Leprosy Hospital, Turkey. Leprosy Review. 1997;68(1):43-9.
24. Mowla MR, Angkur DM, Hasan Z, Sultana MN, Afrin S, Akhter MS. Leprosy patients with deformities at post-elimination stage: The Bangladesh experience. Skin Health and Disease. Skin Health Dis. 2021; 1:e5. https://doi.org/10.1002/ski2.5
25. Ethiraj T, Antony P, Krishnamurthy P, Reddy NBB. A study on the effect of patient and community education in prevention of disability programme. Indian Journal of Leprosy. 1995;67(4):435-45.
26. Sharma P, Kar HK, Beena KR, Kaur H, Narayan R. Disabilities in multibacillary leprosy patients: before, during and after multidrug therapy. Indian Journal of Leprosy. 1996;68(2):127-36.

E. Wrong outcome / study population (n=21)

27. Trautman JR. Care after multidrug therapy. Star. 1993;53(1):7-16.
28. Kuipers M, Schreuders T. The Predictive Value of Sensation Testing in the Development of Neuropathic Ulceration on the Hands of Leprosy Patients. Leprosy Review. 1994;65(3):253-61.
29. Owen BM, Stratford CJ. Assessment of the Methods Available for Testing Sensation in Leprosy Patients in a Rural Setting. Leprosy Review. 1995;66(1):55-62.
30. Smith WCS, Zhang G, Zheng T, Watson JM, Lehman LF, Lever P. Prevention of impairment in leprosy; Results from a collaborative project in China. International Journal of Leprosy. 1995;63(4):507-17.
31. Seboka G, Alert PS. Cost-effective footwear for leprosy control programmes: a study in rural Ethiopia. Leprosy Review. 1996;67(3):208-16.
32. Ghimire M. Secondary deformity in leprosy: a socioeconomic perspective. Asia Pacific Disability Rehabilitation Journal. 2002;13(1):38-44.

33. Abera M, Lema G. The role of support groups in raising the self-concept of people affected by leprosy: an evaluation study in Ethiopia. Asia Pacific Disability Rehabilitation Journal. 2003;14(1):55-62.
34. Abera M, Lemma G. The effectiveness of self-care support groups in the prevention and management of ulcer: an evaluation study in Ethiopia. Asia Pacific Disability Rehabilitation Journal. 2003;14(1):41-54.
35. Knuuttila JP. The fulfilment of health care needs of leprosy patients from Kaski District, Nepal. Leprosy Review. 2004;75(2):153-6.
36. Khadilkar SV, Benny R, Kasegaonkar PS, Khadilkar SV, Benny R, Kasegaonkar PS. Proprioceptive loss in leprous neuropathy: a study of 19 patients. Neurology India. 2008;56(4):450-5.
37. Santos FF, Henriques V, Nhanca M, Seixas J. Orthopedic aspects and disabilities in leprosy patients in Cumura, Guine-Bissau. Tropical Medicine and International Health. 2009;14:173-4.
38. Barreto JG, Salgado CG, Barreto JG, Salgado CG. Clinic-epidemiological evaluation of ulcers in patients with leprosy sequelae and the effect of low level laser therapy on wound healing: a randomized clinical trial. BMC Infectious Diseases. 2010;10(1):237-.
39. Cross H. Prevention of Disability. Leprosy Review. 2010;81(3):254-8.
40. Guerrero MI, Muvdi S, León CI. Delay in leprosy diagnosis as a predictor of disability in a cohort of patients in Colombia, 2000 - 2010. Revista Panamericana de Salud Publica. 2013;33(2):137-43.
41. Cordeiro TL, Frade MAC, Barros A, Foss NT. Baropodometric Evaluations and Sensitivity Alterations in Plantar Ulcer Formation in Leprosy. Int. 2014;13(2):110-5.
42. Shree KLK, Raghu MT, Karinagannanavar A, Manjunatha S. A Study of Proportion of Disability and Its Determinants among Leprosy Patients. J Evol Med Dent Sci-JEMDS. 2015;4(62):10742-6.
43. Veloso Neves T, Díaz Castro JG, Brito de Souza E, Martins Valentim I, Barbosa dos Reis I, Mendes Diniz AP, et al. GRAU DE INCAPACIDADE FÍSICA E ESCORE OLHOS MÃOS -E-PÉS EM PACIENTES HANSÊNICOS PÓS-ALTA. Revista de Atencao Primaria a Saude. 2015;18(3):335-40.
44. de Oliveira MP, de Sousa JR, de Araujo RS, de Sousa Aarão TL, Quaresma JAS. Protein profile of leprosy patients with plantar ulcers from the Eastern Amazon region. Infect [Internet]. 2017 2017/09//; 6(1):[105 p.]. Available from: https://doi.org/10.1186/s40249-017-0318-y
45. Freire de Santana EM, Villaverde Antas EM, Gonçalves de Brito KK, Alves da Silva M. PROFILE OF LEPROSY PATIENTS IN A SECONDARY HEALTH CARE CENTER. Journal of Nursing UFPE / Revista de Enfermagem UFPE. 2017;11(11):4404-9.
46. Mangala H, Jeyaraman M, Chaudhari K, Dhorde V, Likhith D, Murugesh S. A study on prevalence of deformities in leprosy in a tertiary care hospital at davangere. J Mycobact Dis. 2019;9(275):2161-1068.1000275.
47. Govindasamy K, Raja NR, Gupta P, Anand G, Das P, Darlong F, Darlong J. Computer assisted customized footwear and traditional micro-cellular rubber (MCR) footwear to reduce recurrence of ulcer for patients with loss of sensation due to leprosy. Leprosy Review. 2020;91(4):383-92.
